# Yeast Sgf73/Ataxin-7 serves to anchor the deubiquitination module into both SAGA and Slik(SALSA) HAT complexes

**DOI:** 10.1186/1756-8935-2-2

**Published:** 2009-02-18

**Authors:** Kenneth K Lee, Selene K Swanson, Laurence Florens, Michael P Washburn, Jerry L Workman

**Affiliations:** 1 Stowers Institute for Medical Research, E. 50th Street Kansas City, MO 64110, USA

## Abstract

Spinocerebellar ataxia (SCA) is a physically devastating, genetically inherited disorder characterized by abnormal brain function that results in the progressive loss of the ability to coordinate movements. There are many types of SCAs as there are various gene mutations that can cause this disease. SCA types 1–3, 6–10, 12, and 17 result from a trinucleotide repeat expansion in the DNA-coding sequence. Intriguingly, recent work has demonstrated that increased trinucleotde expansions in the SCA7 gene result in defect in the function of the SAGA histone acetyltransferase complex. The *SCA7 *gene encodes a subunit of the SAGA complex. This subunit is conserved in yeast as the *SGF73 *gene. We demonstrate that Sgf73 is required to recruit the histone deubiquitination module into both SAGA and the related SliK(SALSA) complex, and to maintain levels of histone ubiquitination, which is necessary for regulation of transcription at a number of genes.

## Background

Misregulation of transcription is a hallmark of a number of human diseases ranging from diverse classes of cancers to neurodegenerative diseases. Within the organism, the cell uses a number of cues to properly coordinate transcriptional events, including the recruitment of transcriptional coactivator complexes (reviewed in [1]). Many transcriptional coactivator complexes possess intrinsic enzymatic activities that allow for access to DNA for transcription. Chromatin remodeling complexes, such as ATPases, are a type of transcriptional coactivator complex that move nucleosomes, while histone acetyltransferases and histone methyltransferases are examples of complexes that modify histones post-translationally. The importance of these chromatin-modifying complexes is demonstrated by the fact that they are often conserved from yeast to humans (reviewed in [1-3]).

In yeast, one of the most well-characterized processes in regulating chromatin structure is the post-translational modification of histones. Originally identified over 40 years ago, histone acetylation has now become a model for understanding the role of histone modifications in modulating chromatin structure [4]. To this end, the histone acetyltransferase Gcn5 and its associated complexes have been shown to regulate the transcription of up to 10% of genes in yeast [5]. With the advent of high-throughput mass spectrometry, the array of proteins associated with Gcn5 continues to increase [2,6-10]. There are currently at least four Gcn5 complexes: SAGA, SLiK(SALSA), ADA and HAT-A2 [2]. SAGA, the best characterized of these, has 18 protein subunits with distinct functions organized in a modular fashion [1,11,12]. In addition to acetyltransferase activity, a second enzymatic activity has been attributed to SAGA involving the regulation of histone H2B ubiquitination [8,9]. Specifically, the SAGA subunit Ubp8, a deubiquitinating enzyme, has been shown to deubiquitinate H2B, thus allowing for properly regulated transcription at a number of genes. Accentuating the crucial role of modularity within in the SAGA complex, Ubp8 has been shown to require another protein, Sgf11, for its activity [13]. Additionally, Ubp8 and Sgf11 together regulate a subset of genes, and so have been revealed to function as a module in yeast [14]. More recent work has demonstrated that another SAGA subunit, Sus1, is also important for maintaining proper H2B ubiquitination, and that, in fact, it is these three proteins that constitute the deubiquitination module of SAGA and the related SLiK(SALSA) complex [15].

During our study of SAGA we asked how this deubiquitination module is held within the complex. As the initial three-dimensional rendering of SAGA did not contain these proteins, we looked at other components of SAGA. We found that Sgf11 shared a conserved domain with the Sgf73 protein of SAGA, which is implicated in neurodegeneration in higher eukaryotes [16]. Specifically, we discovered that both Sgf11 and Sgf73 contain a conserved non-canonical zinc finger. This zinc finger is termed the ataxin box as it is conserved in the known ataxin proteins [16]. We therefore hypothesized that Sgf73 is related to Sgf11 in function, and found that, indeed, loss of Sgf73 resulted in an increase in global histone ubiquitination. During our studies on Sgf73, the Hurt group also reported the role this Ataxin-7 orthologue has in maintaining proper histone ubiquitination levels in yeast, as well as playing a role in gene gating and mRNA export by recruiting other factors to SAGA [17].

Interestingly, the *SGF73 *gene has been shown to be the *Ataxin-7 *homologue. Mutations in *Ataxin-7 *result in trinucleotide expansions and Spinocerebellur Ataxia-7 (SCA7) [16,18]. SCA7 is one of the many types of spinocerebellar ataxia (SCA), including SCA types 1–3, 6–10, 12, and 17, caused by a genetic defect that results in a trinucleotide repeat expansion in the DNA-coding sequence [19,20]. The fact that *SGF73 *links a histone-modifying complex to a neurodegenerative disorder is intriguing as it was shown that disease levels of trinucleotide expansions of *SCA7 *(36–306 CAG expansions) cause defects in the HAT activity of the SAGA complex *in vitro *[18,21,22]. As diseases of the trinucleotide repeat variety, including Huntington's disease and the SCA disorders, generally exhibit defects in transcription, it is important to note that deletion of *SGF73 *in yeast prevents formation of the transcription pre-initiation complex at a number of genes, and inhibits SAGA recruitment [23].

Here we demonstrate, similar to the Hurt group, that both Sgf11 and Sgf73 are related to the ataxin proteins in higher eukaryotes [17]. Focusing our study on the Gcn5-containing HAT complexes we also looked at the effect of losing Sgf73 on the SAGA-related complex, SliK(SALSA). We found that the deletion of *SGF73 *from SliK(SALSA) also liberates the deubiquitination module from the complex, rendering it non-functional; a similar loss is observed by us and the Hurt group for the SAGA complex [17]. We also observe that Gcn5 HAT activity in SAGA is independent of both Sgf73 and deubiquitination activity. We definitively show that the deubiquitination module of Ubp8, Sgf11 and Sus1 is necessary, but not sufficient to carry out or maintain proper levels of histone H2B ubiquitination in yeast. Additionally, we show that deletion of *SGF73 *in combination with *GCN5 *results in similar defects as the deletion of *UBP8 *and *GCN5*, confirming the importance of the deubiquitination module as part of SAGA for proper transcriptional regulation. Interestingly, the Hurt group showed that in the presence of a fragment of Sgf73 that can co-purify Ubp8, Sgf11 and SusS1, they can observe activity using ubiquitin-AMC hydrolysis [17]. Therefore our data along with the published work from the Hurt lab point to the crucial role that Sgf73 plays in anchoring the deubiquitination module into both SAGA and SliK(SALSA).

## Materials and methods

### Saccharomyces cerevisiae strains

The genotypes of strains used in this study are listed in Table 1. Individual TAP-tagged strains and deletions strains were obtained from Open Biosystems with the exception of strains deleted for *SGF73 *with *K. lactis LEU2*, which were obtained by PCR-mediated knock out as previously described [24]. TAP-tagged strains with deletions were obtained by crossing and dissecting the individual TAP-tagged strain and deletion strain. Deletions and epitope-tagged strains were tested by PCR amplification of genomic DNA or by Western blot analysis.

**Table 1:** *S. cerevisiae *strains.

Name	Genotype	Reference
YKL 117	Mat a his3Δ1 leu2Δ0 met15Δ0 ura3Δ0: Ubp8TAP::HIS3 MX6	Open Biosystems
YKH 045	MATa ura3-1 leu2,3,-112 his3-11,-15 trp1-1 ade2-1 htb1-1 htb2-1 pRS314 [Flag-HTB1-CEN-TRP1] pRG145 [GAPDHprom-3HA-UBI4-URA3 Integrative]	Henry *et al*. 2003
YKH 046	MATa ura3-1 leu2,3,-112 his3-11,-15 trp1-1 ade2-1 htb1-1 htb2-1 pRS314 [Flag-htb1K123R-CEN-TRP1] pRG145 [GAPDHprom-3HA-UBI4-URA3 Integrative]	Henry *et al*. 2003
YKH 047	MATa ura3-1 leu2,3,-112 his3-11,-15 trp1-1 ade2-1 htb1-1 htb2-1 pRS314 [Flag-HTB1-CEN-TRP1] ubp8::KanMx pRG145 [GAPDHprom-3HA-UBI4-URA3 Integrative]	Henry *et al*. 2003
YKL 311	MATa ura3-1 leu2,3,-112 his3-11,-15 trp1-1 ade2-1 htb1-1 htb2-1 pRS314 [Flag-HTB1-CEN-TRP1] sgf73Δ:KAN MX6pRG145 [GAPDHprom-3HA-UBI4-URA3 Integrative]	This Study
YKL 282	Mat a his3Δ1 leu2Δ0 met15Δ0 ura3Δ0: Gcn5TAP::HIS3 MX6; sgf73Δ:KAN MX6	This Study
YKL 281	Mat a his3Δ1 leu2Δ0 met15Δ0 ura3Δ0: Spt8TAP::HIS3 MX6;sgf73Δ:KAN MX6	This Study
YKL 249	Mat a his3Δ1 leu2Δ0 met15Δ0 ura3Δ0: Spt8TAP::HIS3 MX6	Open Biosystem
YKL 306	Mat a his3Δ1 leu2Δ0 met15Δ0 ura3Δ0: Sgf73TAP::HIS3 MX6	Open Biosystem
YKL 301	Mat a his3Δ1 leu2Δ0 met15Δ0 ura3Δ0: Ubp8TAP::HIS3 MX6; sgf73Δ: KANMX6	This Study
YKL 102	Mat a his3Δ1 leu2Δ0 met15Δ0 ura3Δ0: Ada2TAP::HIS3 MX6	Lee *et al*. 2005
YKL 178	Mat a his3Δ1 leu2Δ0 met15Δ0 ura3Δ0: Ubp8TAP::HIS3 MX6; spt20Δ: KANMX6	This Study
YKL 132	Mat a his3Δ1 leu2Δ0 met15Δ0 ura3Δ0: Ubp8TAP::HIS3 MX6; spt20Δ: KANMX6	Lee *et al*. 2005
YKL 128	Mat a his3Δ1 leu2Δ0 met15Δ0 ura3Δ0: Ada2TAP::HIS3 MX6; sgf11Δ:KANMX6	Lee *et al*. 2005
FY 2033	Mata HA-Spt7-1180-TAP:TRP1 ura3Δ0 leu2Δ1 trp1Δ63 his4-917δlys-173R2	Wu and Winston, 2002
YKL 151	Mat a his3Δ1 leu2Δ0 met15Δ0 ura3Δ0: gcn5Δ::KAN MX6	Open Biosystems
YKL 297	Mat a his3Δ1 leu2Δ0 met15Δ0 ura3Δ0: sgf73Δ::KAN MX6	Open Biosystems
By4741	Mat a his3Δ1 leu2Δ0 met15Δ0 ura3Δ0	Open Biosystems
YKL 364	Mat a his3Δ1 leu2Δ0 met15Δ0 ura3Δ0: Ada2TAP::HIS3 MX6; sgf73Δ:KAN MX6	This Study
YKL 357	Mata HA-Spt7-1180-TAP:TRP1; sgf73 Δ:KAN MX6 ura3Δ0 leu2Δ1 trp1Δ63 his4-917δlys-173R2	This Study
YKL 135	Mat a his3Δ1 leu2Δ0 met15Δ0 ura3Δ0: gcn5Δ::KAN MX6; ubp8Δ::KAN MX6	Lee *et al*. 2005
YKL 137	Mat a his3Δ1 leu2Δ0 met15Δ0 ura3Δ0: sgf11Δ::KAN MX6; ubp8Δ::KAN MX6	Lee *et al*. 2005
YKL 358	Mat a his3Δ1 leu2Δ0 met15Δ0 ura3Δ0: sgf73Δ::KAN MX6; gcn5Δ::KAN	This Study
YKL 360	Mat a his3Δ1 leu2Δ0 met15Δ0 ura3Δ0: sgf73Δ::LEU2; ubp8Δ::KAN MX6	This Study

Phenotypic analysis of the various yeast strains was carried out using serial dilutions of saturated cultures. Serial dilutions were carried out by spotting 10 μl of ~ 1.85 × 10^7 ^cells/ml (OD_600 _= 1.0) and diluting four-fold for each spot onto either YP + dextrose or YP + galactose plates. Plates were imaged after two days of growth at 30°C.

### Plasmid construction

The *SGF73 *promoters and coding sequences were amplified from yeast genomic DNA using primers containing a 5'*Sma*I site and a 3' *Sma*I site (primer sequences available upon request). This PCR product was cloned in-frame into a vector containing a c-terminal HA tag (pBL525). Subsequently, the resultant plasmid was mutated using the Quickchange (Stratagene) mutagenesis system to create a *Sma*I site in the 5' end of the gene and eliminate the *Sma*I site in the 5' end of the promoter to allow for movement of ORFs in and out of the resulting pBL525-SGF73PRO vector. PCR products were initially cloned into pGEM T-Easy vector (Promega, Madison, WI).

### TAP purification

Purification of TAP-tagged complexes was carried out as previously described [25] with the following modifications: Elutions were carried out in a volume of 500 μl and repeated five times for a total volume of 3 ml of purified complexes, which was concentrated six-fold with Amicon Ultra concentrators with a MW cutoff of 100 kilodaltons. Purifications of complexes containing pBL525 plasmids were carried out from cultures grown in media lacking leucine to maintain selection for the various plasmids used in this study. TAP-purified complexes were resolved on a 10% SDS-PAGE gel and visualized by silver staining.

### Multidimensional Protein Identification Technology (MudPIT) analysis

TCA-precipitated proteins were urea-denatured, reduced, alkylated and digested with endoproteinase Lys-C (Roche) followed by modified trypsin (Promega) as described in [26]. Peptide mixtures were loaded onto 100 μm fused silica microcapillary columns packed with 5 μm C_18 _reverse phase (Aqua, Phenomenex), strong cation exchange particles (Partisphere SCX, Whatman), and reverse phase [27]. Loaded microcapillary columns were placed in-line with a Quaternary 1100 series HPLC pump (Agilent) and a LTQ or XP linear ion trap mass spectrometer equipped with a nano-LC electrospray ionization source (ThermoFinnigan). Fully automated 10-step MudPIT runs were carried out on the electrosprayed peptides, as described in [28]. Tandem mass (MS/MS) spectra were interpreted using SEQUEST [29] against a database of 11,982 amino acid sequences, consisting of 5877 *S. cerevisiae *proteins (non-redundant entries from NCBI 2007-03-14 release), 177 usual contaminants (such as human keratins, IgGs, and proteolytic enzymes), and, to estimate false discovery rates (FDR), 5877 randomized sequences for each non-redundant protein entry. Peptide/spectrum matches were selected and compared using DTASelect/CONTRAST [30] with the following criteria set: spectra/peptide matches were only retained if they had a DeltCn of at least 0.08, and minimum XCorr of 1.8 for singly-, 2.5 for doubly-, and 3.5 for triply-charged spectra. In addition, peptides had to be fully tryptic and at least seven amino acids long. Combining all runs, proteins had to be detected by at least two such peptides, or one peptide with two independent spectra. Under these criteria, the FDR ranges from 0 to 0.1606% (Additional file 1). To estimate relative protein levels, normalized spectral abundance factors (NSAFs) were calculated for each non-redundant protein, as described in [31,32].

### Isolation of ubiquitylated histones

The relative levels of ubiquitinated histone H2B (ubH2B) in different strain backgrounds (YKH045, YKH046, YKH047, YKL142 and YKL311) were determined by purifying histone H2B using an N-terminally Flag-tagged histone H2B and subsequently detecting the different forms of H2B using an anti-Flag-HRP antibody (Sigma) as described previously [33]. Purifications of ubH2B substrate for the deubiquitination assays described below were also carried out as described above.

### Deubiquitination assay

The Flag-tagged H2B substrate (containing ubH2B and unmodified H2B) was obtained as described [33]. Between 250 and 500 ng of this substrate was incubated at 30°C for 60 min in DUB buffer (10 mM Tris-HCl at pH 8.0, 1 mM DTT, 1 μM PMSF, 1 μg/mL aprotinin and pepstatin A) with equal amounts of concentrated TAP-purified complexes. As a control, substrate was also incubated in DUB buffer to which only calmodulin elution buffer was added. The reaction was stopped by freezing in liquid nitrogen, followed by boiling in one volume of 2× SDS Sample Buffer for 5 min, and running on a 15% SDS-PAGE gel. Gels were transferred to PVDF membrane (Immobilon), and Western blot analysis was performed using anti-Flag-HRP (to detect ubH2B and H2B) and anti-HA-HRP (to detect ubiquitin) antibodies.

### HAT assays

HAT assays were carried out as previously described [34].

## Results

### Yeast Ataxin-7 is required to anchor the histone deubiquitination module into SAGA

**Figure 1:**
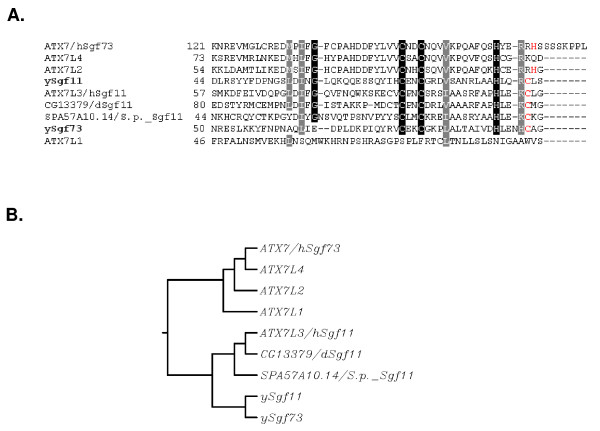
**Evolutionary conservation of *SGF73 *and *SGF11***. A. Alignment of *S. cerevisiae*, *S. pombe*, *D. melanogaster*, *H. sapien *orthologues of Ataxin7 reveals the conservation of the Ataxin block, zinc finger(s). Conserved residues are indicated in black and semi-conserved residues in gray. B. Phylogenetic analysis revealed that in addition to *SGF73, SGF11 *is also a member of the *Ataxin-7 *family of genes.

**Figure 2:**
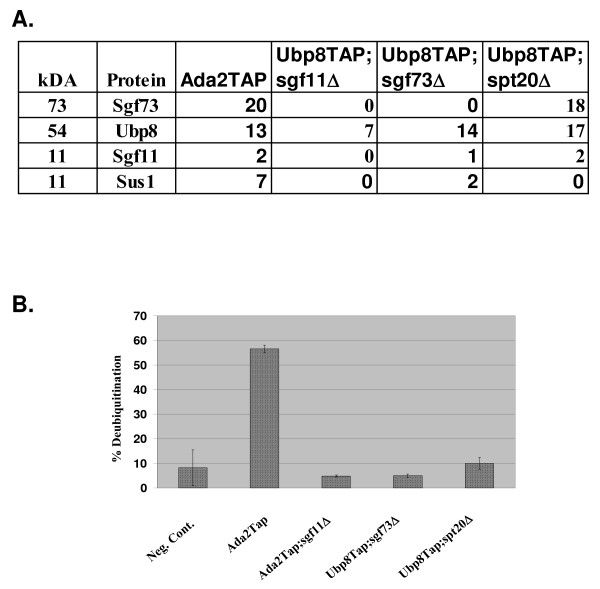
**Analysis of Ubp8 purifications in the absence of *SGF73***. A. Mudpit analysis of various Ubp8Tap purifications reveals the effect of the loss of *SGF73 *and *SPT20 *versus *SGF11 *on Ubp8-associated complexes. In the absence of *SGF73*, Ubp8 only co-purifies with other deubiquitination module components, Sus1 and Sgf11. The numbers represent total peptides obtained for each component. B. Ubp8-associated complexes purified in the absence of *SGF73 *and *SPT20 *lose the ability to deubiquitinate histone H2B *in vitro*.

Our previous work, along with the work of others, has identified the core deubiquitination module with the SAGA/SLiK(SALSA) complex to be composed of Ubp8, Sgf11 and Sus1 [13-15]. We also demonstrated that Ubp8 needed to be part of SAGA in order to carry out deubiquitination of histone H2B. This led us to determine which component of SAGA was required to anchor this module into the SAGA complex. Phylogenetic and bioinformatic studies revealed that the yeast *Ataxin-7 *gene *SGF73 *was related to *SGF11 *in that they both contain conserved Ataxin box zinc-fingers (Figure 1A). We also observed that *SGF11 *and *SGF73 *are more closely related to each other than to the other Ataxin orthologues (Figure 1B and see discussion). This discovery led us to hypothesize that Sgf73 may also function in regulating deubiquitination in yeast. We first tested this by purifying Ubp8-associated complexes in the absence of *SGF73*. Using TAP-tag purification followed by MudPit analysis we found that Ubp8 was only associated with the other core deubiquitination module members, Sgf11 and Sus1, upon the deletion of *SGF73 *(Figure 2A). This is the first time the entire deubiquitination module was isolated *in vivo*. In order to validate our mass spectrometry results that Gcn5 was lost from this complex we tested the purified mutant complexes for acetyltransferase activity and found that this activity is indeed lost in each of these purifications (data not shown). This led us to revisit the question of whether this module, now complete, could deubiquitinate on its own in the absence of an association with SAGA. We found that, similar to Ubp8 complexes purified in the absence of Sgf11, Ubp8 complexes purified in the absence of *SGF73 *could not deubiquitinate H2B *in vitro *(Figure 2B) [13,14]. During the preparation of this manuscript, the Hurt group demonstrated that Ubp8, Sgf11, Sus1 and an N-terminal fragment of Sgf73 could hydrolyze ubiquitin in a fluorescence-based assay and therefore was the minimal set of proteins required for deubiquitination activity [15].

### Yeast Ataxin-7 is required to maintain proper histone ubiquitination levels in Saccharomyces cerevisiae

**Figure 3:**
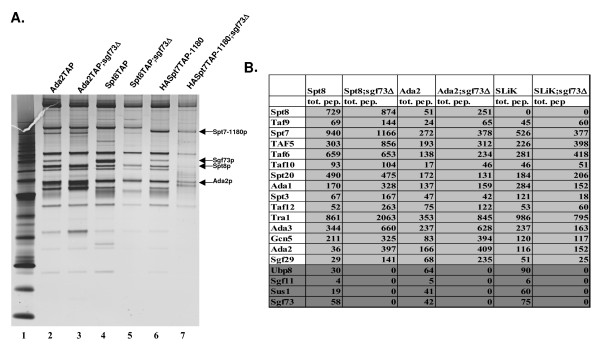
**Deletion of *SGF73 *results in the loss of the deubiquitination module from the SAGA/SLiK(SALSA) complexes**. A. Silver stain of various SAGA/SLiK(SALSA) purifications. Lane 1 marker, Lane 2 Ada2Tap purification, Lane 3 Ada2TAP;sgf73Δ purification, Lane 4 Spt8Tap purification, Lane 5 Spt8TAP;sgf73Δ purification, Lane 6 SLiK(SALSA) purification from an HASpt7-1180TAP strain, where Spt7 lacks the C-terminus required for Spt8 association, Lane 7 HASpt7-1180TAP; sgf73Δ. The subunits that were used for purification are labeled, as well as the band corresponding to Sgf73. B. MudPit analysis reveals that deletion of *SGF73 *from Spt8, Gcn5 and HA-Spt7-1180TAP (Slik) purifications results in the loss of the deubiquitination module, ubp8, sgf11 and Sus1 from SAGA (numbers are total peptides obtained for each subunit).

In order to understand our initial findings about *SGF73/Ataxin7 *using a Ubp8TAP tag strain, we decided to delete *SGF73 *from a variety of yeast strains, which would allow us to answer a number of additional questions. The strains from which we deleted *SGF73 *were: SPT8TAP, which purifies only SAGA, HA-SPT7-1180TAP, which purifies only Slik(SALSA), ADA2TAP and GCN5TAP to purify all Gcn5-containing complexes. From these purifications we could determine that in general the complexes remained intact. In order to determine if specific subunits were lost we performed MudPit analysis (Figure 3A and Figure S1A). Our MudPit analysis again revealed that the role of Sgf73/Ataxin-7 was indeed to tether the deubiquitination module into both the SAGA and SLiK(SALSA) complexes (Figure 3B and Figure S1B).

**Figure 4:**
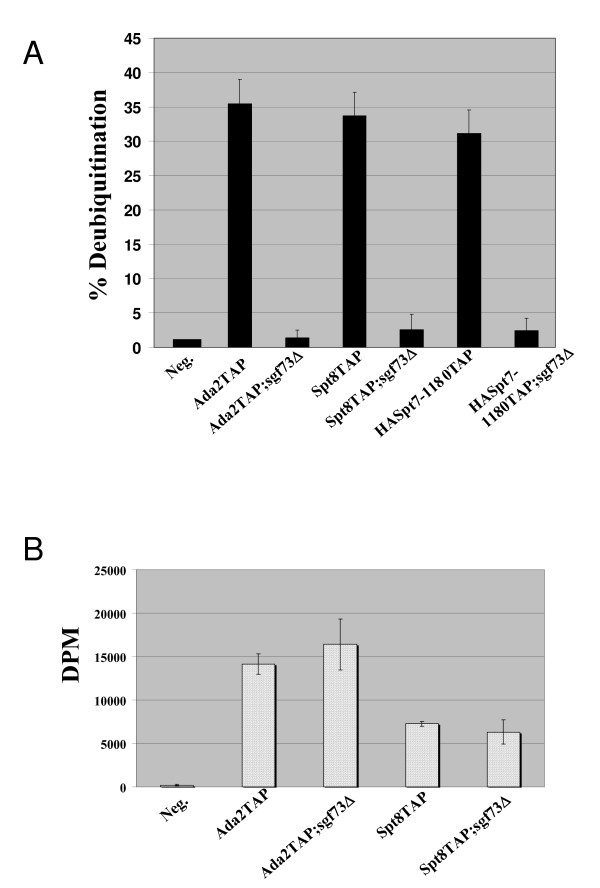
**Loss of SGF73 leads to the loss of *in vitro *histone deubiquitination activity, but does not affect HAT activity**. A. Quantification of deubiquitination activity associated with various Gcn5-containing HAT complexes in the presence or absence of Sgf73. B. Quantification of the histone acetyltransferase activity associated with various Gcn5-containing HAT complexes in the presence of absence of Sgf73.

We next tested whether these complexes could deubiquitinate H2B *in vitro*. None of the complexes purified in the absence of *SGF73 *were able to deubiquitinate H2B *in vitro *(Figure 4A). We also demonstrated for the first time that the SLiK(SALSA) complex, purified using a truncation of Spt7, deubiquitinates H2B and also requires *SGF73 *for maintenance of deubiquitination activity [35] (Figure 3B and Figure 4A). We then tested whether deletion of *SGF73 *affects the HAT activity of SAGA or SliK(SALSA), and found that acetyltransferase activity is essentially unaffected in a *SGF73 *deletion (Figure 4B). In order to further validate the importance of *SGF73 *for the maintenance and regulation of histone deubiquitination in yeast, we next determined if the addition of a plasmid expressing Sgf73 into the *SGF73 *deletion strains could restore the deubiquitination module to the complexes and subsequently rescue the deubiquitination activity of the purified SAGA/SliK complexes. MudPit analysis of this complex shows that the plasmid-expressed Sgf73-HA rescues incorporation of Sgf73 into SAGA/SLiK(SALSA) and also recovered Ubp8, Sgf11 and Sus1, suggesting that the complex would again be functional for deubiquitination (Additional file 2B). In order to confirm that this rescued complex regained the ability to deubiquitinate H2B *in vitro*, we tested it in our deubiquitination assay and found that indeed, the rescued complex partially restored activity compared with the complex purified in the presence of an empty plasmid (Additional file 2C; compare lane 3 to 4 and D).

**Figure 5:**
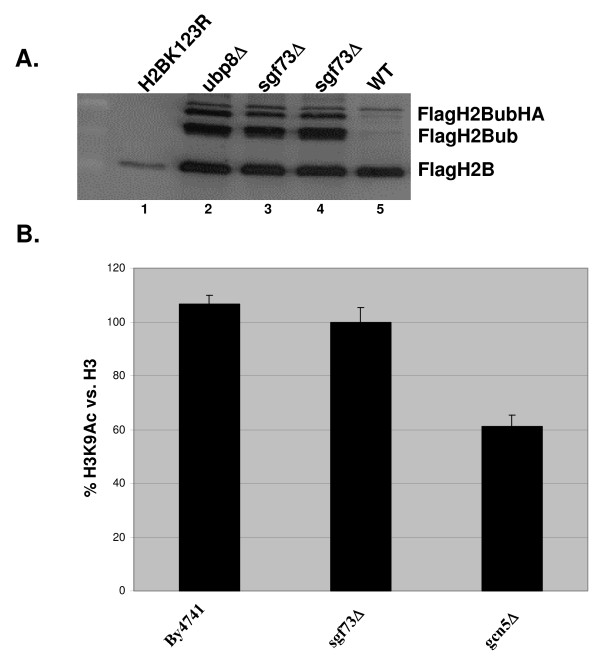
**Deletion of *SGF73 *results in a global increase in H2B ubiquitin levels similar to deletion of *UBP8*, but does not affect H3 acetylation**. A. Flag western blot analysis of H2Bub levels: Lane 1 H2BK123R, Lane 2 UBP8Δ, Lane 3 SGF73Δ, Lane 4 SGF73Δ, Lane 5 wildtype. The indicated bands represent FlagH2B, FlagH2Bub and FlagH2BubHA. B. Quantification of H3 lysine 9 Western blot signal demonstrates that deletion of *SGF73 *does not reduce global histone H3 lysine 9 acetylation when compared with deletion of *GCN5*.

We next examined the effect on global histone ubiquitination of deleting *SGF73*. Deletion of *SGF73 *from a Flag-tagged histone H2B strain causes an increase in H2B ubiquitination similar to deletion of *UBP8 *(Figure 5A; compare lane 2 with lanes 3 and 4). Previous reports using chromatin immunoprecipitation demonstrated that loss of *SGF73 *caused local defects in pre-initiation complex (PIC) assembly and acetylation [23]. In order to determine if the global defects in histone modification levels caused by deletion of *SGF73 *were limited to histone ubiquitination, we also checked whether histone H3 acetylation was affected by this deletion. We found that deletion of *SGF73 *had no discernable affect on the global levels of H3K9 acetylation (Figure 5B).

**Figure 6:**
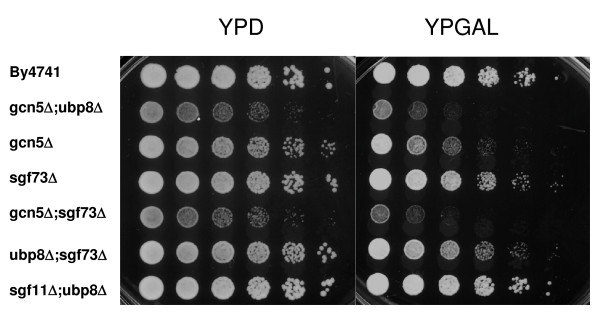
**Similar to a *UBP8 *deletion, deletion of SGF73 in combination with *GCN5 *results in a GAL phenotype**. Cell growth of the sgf73Δ in combination with gcn5Δ or ubp8Δ. Four-fold serial dilutions were spotted on plates containing rich medium with dextrose (YPD) or with galactose (YP-Gal). Wildtype and mutants were grown at 30°C for ~ 2 days.

Previous work demonstrated that the combined loss of acetylation and deubiquitination through the deletion of *GCN5 *and *UBP8 *resulted in transcriptional defects in yeast [8,13,14]. In particular, the ability to utilize galactose as a carbon source is compromised in yeast deleted for *GCN5 *in combination with *UBP8 *or *SGF11*. Therefore, we hypothesized that deletion of *SGF73 *in combination with *GCN5 *would result in a similar defect. However, yeast with the deletion of *SGF73 *in combination with another component of the deubiquitination module should be able to grow in the presence of galactose as the sole carbon source. Figure 6 demonstrates that this is indeed the case, as deletion of *SGF73 *in combination with *GCN5 *shows a similar phenotype to yeast deleted for *UBP8 *and *GCN5*, while deletion of *SGF73 *with *UBP8 *grows similar to wildtype and more importantly, similarly to yeast deleted for *UBP8 *and *SGF11*.

## Discussion

In this study we identify a role for the yeast orthologue of *Ataxin-7, SGF73*, in maintaining proper histone ubiquitination levels in yeast by the anchoring the histone deubiquitination module into the SAGA and SLiK(SALSA) complex. Our initial hypothesis about the potential role for *SGF73 *in deubiquitination came from the observation that like *SGF73 *and other ataxin genes, *SGF11 *also contained a signature ataxin-box zinc finger motif. Since Sgf11 had a role in maintaining the integrity of the deubiquitination module in SAGA, we reasoned that these domains may help mediate the association. We and others observed that while Ataxin-7 in humans contains three ataxin boxes, yeast SGF73 only contains two ataxin boxes, with a third ataxin box residing in Sgf11 [16]. Therefore the yeast SAGA complex contains three ataxin boxes, while the human STAGA complex contains the three from Ataxin-7 and two additional ones from the human Sgf11, Ataxin 7L3.

Sgf73 was originally identified as a SAGA-factor of 73 kilodaltons via its association with Gcn5 [6]. We demonstrate that Sgf73 is part of both the SAGA and SLiK(SALSA) complex, but is not present in the other known Gcn5 complex, ADA (data not shown). We show that deletion of *SGF73 *results in the loss of all three subunits of the deubiquitination module in SAGA, Ubp8, Sgf11 and Sus1. This effect differs from the deletion of *UBP8, SGF11 *or *SUS1*, which result only in pairwise loss of the other members of the module [13-15]. In addition, overexpression of Sgf73 in our deletion strain partially rescues defects that were observed in the *SGF73 *deletion; namely restoring incorporation of the deubiquitination module into SAGA and thus partially restoring the ability of SAGA to deubiquitinate H2B *in vitro* (Figure S2). A possible reason why we could not fully restore deubiquitination activity in our rescue experiment is that the rescued complex is purified under selective conditions, which alters the amount of SAGA and Slik(SALSA) in the cell (Lee and Workman, unpublished). Our finding demonstrates that Sgf73 is vital for the association of the deubiquitination module in SAGA and SliK(SALSA). We also show that although we can purify the deubiquitination module away from SAGA and SliK(SALSA) by deleting *SGF73 *in a Ubp8-tagged strain, this sub-complex is unable to deubiquitinate histone H2B *in vitro*, further verifying the previous finding that Ubp8 requires association with SAGA in order to carry out its function.

Although Sgf73 was previously shown to be important for PIC formation and histone acetylation at a number of genes, we observed that the global landscape of H3 acetylation is unaffected by deletion of *SGF73*. This further lends support to the idea that the acetylation and deubiquitination modules of SAGA are separable in function.

In humans Ataxin-7 is part of the STAGA histone acetyltransferase complex. Previous reports have shown that polyglutamate-expanded Ataxin-7 containing 92 repeats results in a partial disruption and subsequent defect in histone acetylation. This observation has been seen in both human and yeast cells [18,21]. However, there is controversy surrounding this finding as others have reported that the polyglutamate-expanded Ataxin-7 does not have this effect [22]. At the time, these studies did not take into account histone deubiquitination, as the role of Ataxin-7/Sgf73 in this process was not yet appreciated. It will be interesting to revisit the effect that these trinucleotide expanded proteins have on SAGA deubiquitination activity, as well as the ability of these proteins to re-establish the deubiquitination module within SAGA. If polyglutamate expansions do indeed affect these processes, analysis of histone ubiquitination levels could be a diagnostic tool for looking at SCA7 disease states.

## Competing interests

The authors declare that they have no competing interests.

## Authors' contributions

KKL conceived of the experiments, carried out the genetic and biochemical studies and drafted the manuscript. SKS, LF and MPW performed the mass spectrometry and preliminary analysis, and JLW conceived of the study, participated in its design and coordination and helped to draft the manuscript. All authors read and approved the final manuscript.

## Supplementary Material

Additional file 1 **Figure S1 A.** Silver stain of various SAGA/SLiK(SALSA) purifications. Lane 1 marker, Lane 2 Spt8Tap purification, Lane 3 Gcn5TAP;sgf73Δ purification, Lane 4 Gcn5Tap purification, Lane 5 Spt8TAP;sgf73Δ purification, Lane 6 SLiK(SALSA) purification from an Spt7TAP strain, where Spt7 lacks the C-terminus required for Spt8 association, Lane 7 Sgf73TAP purification B. MudPit analysis of Sgf73TAP purification compared to the Gcn5TAP purifications in the presence or absence of SGF73. Click here for file

Additional file 2 **Figure S2 Description: Sgf73 provided in trans is able to incorporate into the SAGA/SLiK (SALSA) complex and partially rescue histone deubiquitination activity.** A. Silver stain gel showing purification of SAGA/SLiK(SALSA) from yeast expressing SGF73 from a vector under control of it's own promoter (Lane 1). B. MudPit analysis of the rescued complex compared with the complex purified with only an empty vector. Highlighted rows indicate the proteins that are recruited back into the complex after the addition of Sgf73. C.*In vitro *deubiquitination assay demonstrating that the rescued complex is able to partially rescue the ability to deubiquitinate histone H2B, Lane 1 negative control, Lane 2 Spt8TAP, Lane 3 Gcn5Tap;sgf73Δ + empty vector, Lane 4 Gcn5Tap;sgf73Δ + SGF73. D. Quantification of data obtained in C. Click here for file
