# Supplementary figures and images for: Yeast Sgf73/Ataxin-7 serves to anchor the deubiquitination module into both SAGA and Slik(SALSA) HAT complexes
Source: Epigenetics Chromatin. 2009 Feb 18;2:2. doi: 10.1186/1756-8935-2-2 (PMC2657900; doi:10.1186/1756-8935-2-2)

## Slide 1
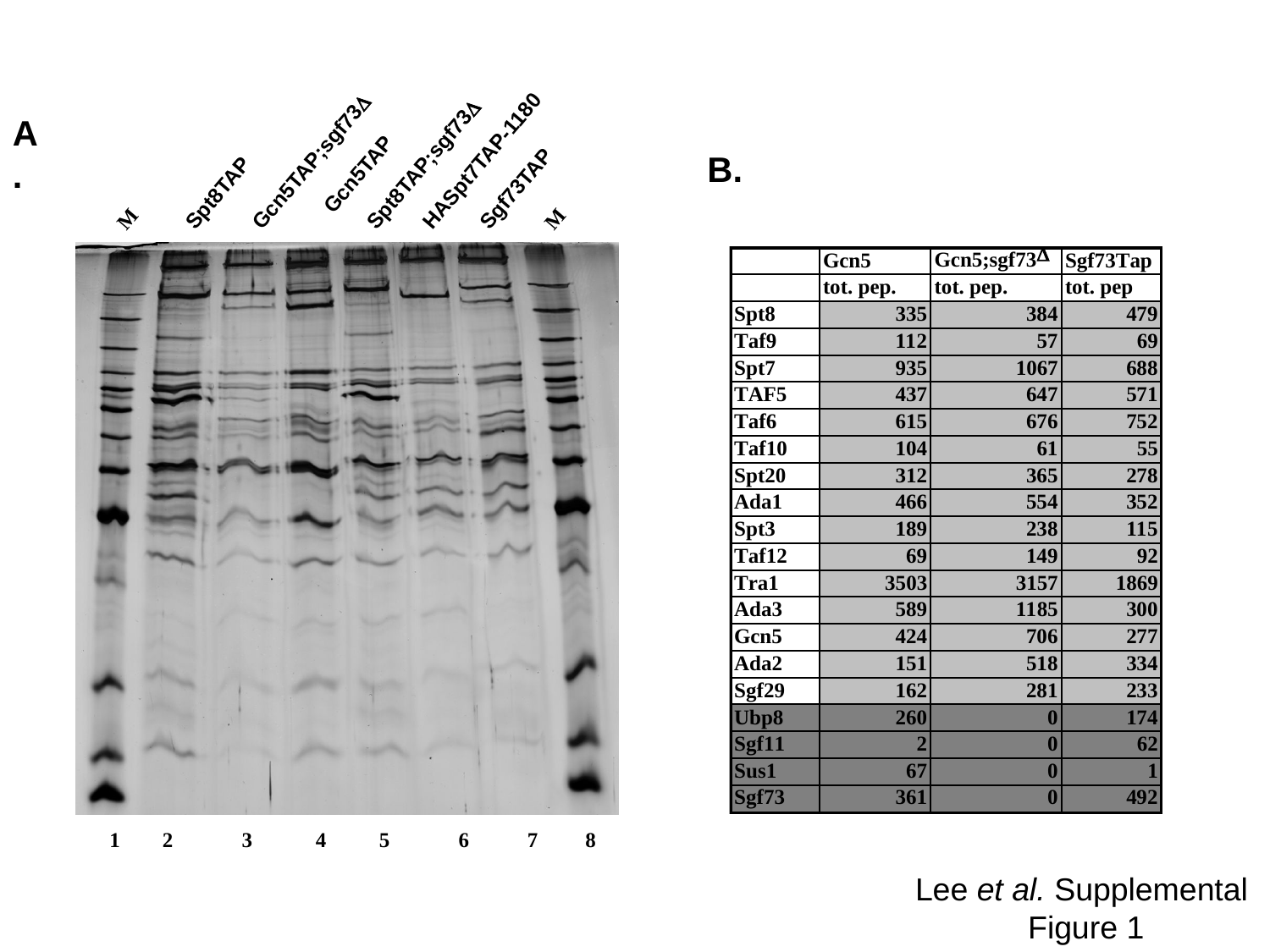

# A.
B.
HASpt7TAP-1180
Gcn5TAP;sgf73Δ
Spt8TAP;sgf73Δ
Gcn5TAP
Sgf73TAP
Spt8TAP
M
M
1 2 3 4 5 6 7 8
Lee et al. Supplemental
Figure 1

Supplement: Additional file 1 — Figure S1 A. Silver stain of various SAGA/SLiK(SALSA) purifications. Lane 1 marker, Lane 2 Spt8Tap purification, Lane 3 Gcn5TAP;sgf73Δ purification, Lane 4 Gcn5Tap purification, Lane 5 Spt8TAP;sgf73Δ purification, Lane 6 SLiK(SALSA) purification from an Spt7TAP strain, where Spt7 lacks the C-terminus required for Spt8 association, Lane 7 Sgf73TAP purification B. MudPit analysis of Sgf73TAP purification compared to the Gcn5TAP purifications in the presence or absence of SGF73. [file 1756-8935-2-2-S1.ppt]
