# Supplementary material for: Yeast Sgf73/Ataxin-7 serves to anchor the deubiquitination module into both SAGA and Slik(SALSA) HAT complexes
Source: Epigenetics Chromatin. 2009 Feb 18;2:2. doi: 10.1186/1756-8935-2-2 (PMC2657900; doi:10.1186/1756-8935-2-2)
Supplement: Additional file 2 — Figure S2 Description: Sgf73 provided in trans is able to incorporate into the SAGA/SLiK (SALSA) complex and partially rescue histone deubiquitination activity. A. Silver stain gel showing purification of SAGA/SLiK(SALSA) from yeast expressing SGF73 from a vector under control of it's own promoter (Lane 1). B. MudPit analysis of the rescued complex compared with the complex purified with only an empty vector. Highlighted rows indicate the proteins that are recruited back into the complex after the addition of Sgf73. C.In vitro deubiquitination assay demonstrating that the rescued complex is able to partially rescue the ability to deubiquitinate histone H2B, Lane 1 negative control, Lane 2 Spt8TAP, Lane 3 Gcn5Tap;sgf73Δ + empty vector, Lane 4 Gcn5Tap;sgf73Δ + SGF73. D. Quantification of data obtained in C. [file 1756-8935-2-2-S2.ppt]

## Slide 1
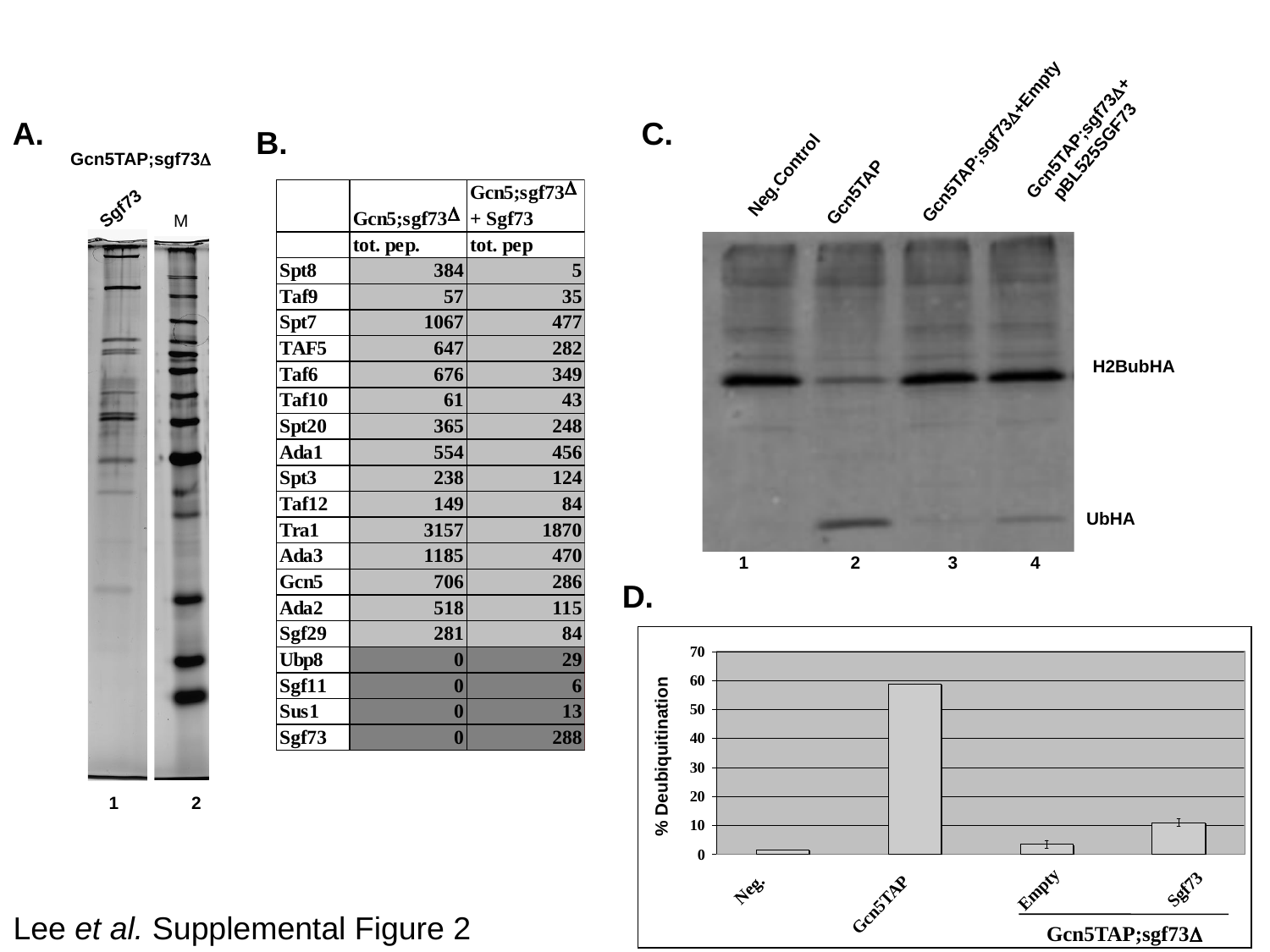

# B.
A.
C.
Gcn5TAP;sgf73Δ+
pBL525SGF73
Gcn5TAP;sgf73Δ+Empty
Gcn5TAP;sgf73Δ
Neg.Control
Gcn5TAP
Sgf73
M
H2BubHA
UbHA
1 2 3 4
D.
% Deubiquitination
 1 2
Empty
Sgf73
Neg.
Gcn5TAP
Lee et al. Supplemental Figure 2
Gcn5TAP;sgf73Δ
